# Prevalence of Influenza A viruses in wild migratory birds in Alaska: Patterns of variation in detection at a crossroads of intercontinental flyways

**DOI:** 10.1186/1743-422X-5-71

**Published:** 2008-06-04

**Authors:** Hon S Ip, Paul L Flint, J Christian Franson, Robert J Dusek, Dirk V Derksen, Robert E Gill, Craig R Ely, John M Pearce, Richard B Lanctot, Steven M Matsuoka, David B Irons, Julian B Fischer, Russell M Oates, Margaret R Petersen, Thomas F Fondell, Deborah A Rocque, Janice C Pedersen, Thomas C Rothe

**Affiliations:** 1 US Geological Survey, National Wildlife Health Center, Madison, Wisconsin, USA; 2 US Geological Survey, Alaska Science Center, Anchorage, Alaska, USA; 3 US Fish and Wildlife Service, Anchorage, Alaska, USA; 4 US Department of Agriculture, National Veterinary Services Laboratory, Ames, Iowa, USA; 5 Alaska Department of Fish and Game, Anchorage, Alaska, USA

## Abstract

**Background:**

The global spread of the highly pathogenic avian influenza H5N1 virus has stimulated interest in a better understanding of the mechanisms of H5N1 dispersal, including the potential role of migratory birds as carriers. Although wild birds have been found dead during H5N1 outbreaks, evidence suggests that others have survived natural infections, and recent studies have shown several species of ducks capable of surviving experimental inoculations of H5N1 and shedding virus. To investigate the possibility of migratory birds as a means of H5N1 dispersal into North America, we monitored for the virus in a surveillance program based on the risk that wild birds may carry the virus from Asia.

**Results:**

Of 16,797 birds sampled in Alaska between May 2006 and March 2007, low pathogenic avian influenza viruses were detected in 1.7% by rRT-PCR but no highly pathogenic viruses were found. Our data suggest that prevalence varied among sampling locations, species (highest in waterfowl, lowest in passerines), ages (juveniles higher than adults), sexes (males higher than females), date (highest in autumn), and analytical technique (rRT-PCR prevalence = 1.7%; virus isolation prevalence = 1.5%).

**Conclusion:**

The prevalence of low pathogenic avian influenza viruses isolated from wild birds depends on biological, temporal, and geographical factors, as well as testing methods. Future studies should control for, or sample across, these sources of variation to allow direct comparison of prevalence rates.

## Background

Wild aquatic birds are considered the reservoir for all subtypes of influenza A viruses, with most infections thought to be inapparent [1]. Bird-to-bird and bird-to-mammal transmission may result in the establishment of influenza viruses in new hosts, with some possibly evolving into highly pathogenic avian influenza (HPAI) viruses in poultry and pandemic influenza viruses in humans [2]. Outbreaks of HPAI H5N1 virus in wild birds have been associated with mortality [3,4]. However, reports that apparently healthy wild birds are infected with HPAI H5N1 [5-7] substantiate concerns that birds may distribute this virus during migration [3,8,9]. The ongoing debate on the role of wild birds in the spread of HPAI H5N1 highlights the need for information on both avian influenza (AI) viruses in migratory birds, and on life history of birds (e.g., distribution and behavior) and their linkage in terms of AI transmission [10,11].

Significant numbers of birds migrate between Asia, where the HPAI H5N1 virus is endemic, and high latitude areas of North America. It is estimated that 1.5–2.3 million birds migrate from Asia to Alaska each year [12]. While the existence of distinct North American and Eurasian AI lineages indicates that large-scale transmission of AI viruses between regions does not occur [1,13], examples of intercontinental transmission exist [14,15]. Such exchange likely occurs where migratory paths of continental populations cross [16]. Given the current distribution of HPAI H5N1, Alaska is likely to be one of the first locations where this virus, if introduced by migratory birds, will occur in North America.

In 2006, an interagency strategic plan was developed by the US Department of the Interior (DOI), US Department of Agriculture (USDA), and state, tribal, and other organizations to conduct monitoring for early detection of the introduction of HPAI H5N1 into North America by migratory birds [17]. The US Geological Survey (USGS), US Fish and Wildlife Service (USFWS), and partners developed an AI surveillance sampling plan for Alaska based on a detailed assessment of migratory bird distribution and ecology and the global HPAI H5N1 epizootiological situation. Here, we present the DOI sampling plan for Alaska and our findings of AI virus prevalence by species, location, age, sex, and date. We discuss our findings of low-pathogenic AI viruses in the context of sources of variation in prevalence and future sampling designs for monitoring and detection of avian influenza viruses.

## Results

### Avian influenza prevalence overview

Samples from 16,797 birds were collected between May 2006 and March 2007 [see additional file 1: Results of avian influenza surveillance in Alaska between May 2006 and March 2007], including 5,111 samples from hunter-harvested birds (n = 4,358 from the subsistence hunt, prior to 1 July; n = 753 from the autumn hunt, after 1 August) and 11,686 samples from live wild birds. Cloacal swabs accounted for 90.7% (n = 15,231) of the samples and 9.3% (n = 1,566) were fresh fecal samples. A total of 126 species of birds were tested, comprising 10 orders and 27 families. We obtained 200 or more samples from 17 of the 26 priority species.

A total of 293 samples were positive in the matrix rRT-PCR test for an overall AI prevalence of 1.7% (CI 1.5–2.1%). These 293 samples, as well as 12,149 matrix rRT-PCR negatives (for a total of 12,442 or 74.1% of all samples collected) were further tested by virus isolation. AI viruses were isolated from 189 of these samples. Overall AI prevalence based on virus isolation was 1.5% (CI 1.1–3.5%) and was significantly different from our *a priori *expectation (Ho: VI% > rRT-PCR%, 1 tailed t_α = 0.05 _= -1.51, *P *< 0.05).

The rRT-PCR and virus isolation tests agreed (either positive or negative) for 97.5% (CI 96–98.5%) of the samples. Of the samples that were positive on the initial rRT-PCR test, 54.8% (n = 155, CI 48.9–60.8%) were negative on virus isolation. Conversely, of the samples that were negative on the initial rRT-PCR test, 0.5% (n = 61, CI 0.4–0.6%) were positive based on virus isolation. No highly pathogenic viruses were identified using either rRT-PCR or virus isolation.

### Geographic variation

**Figure 1:**
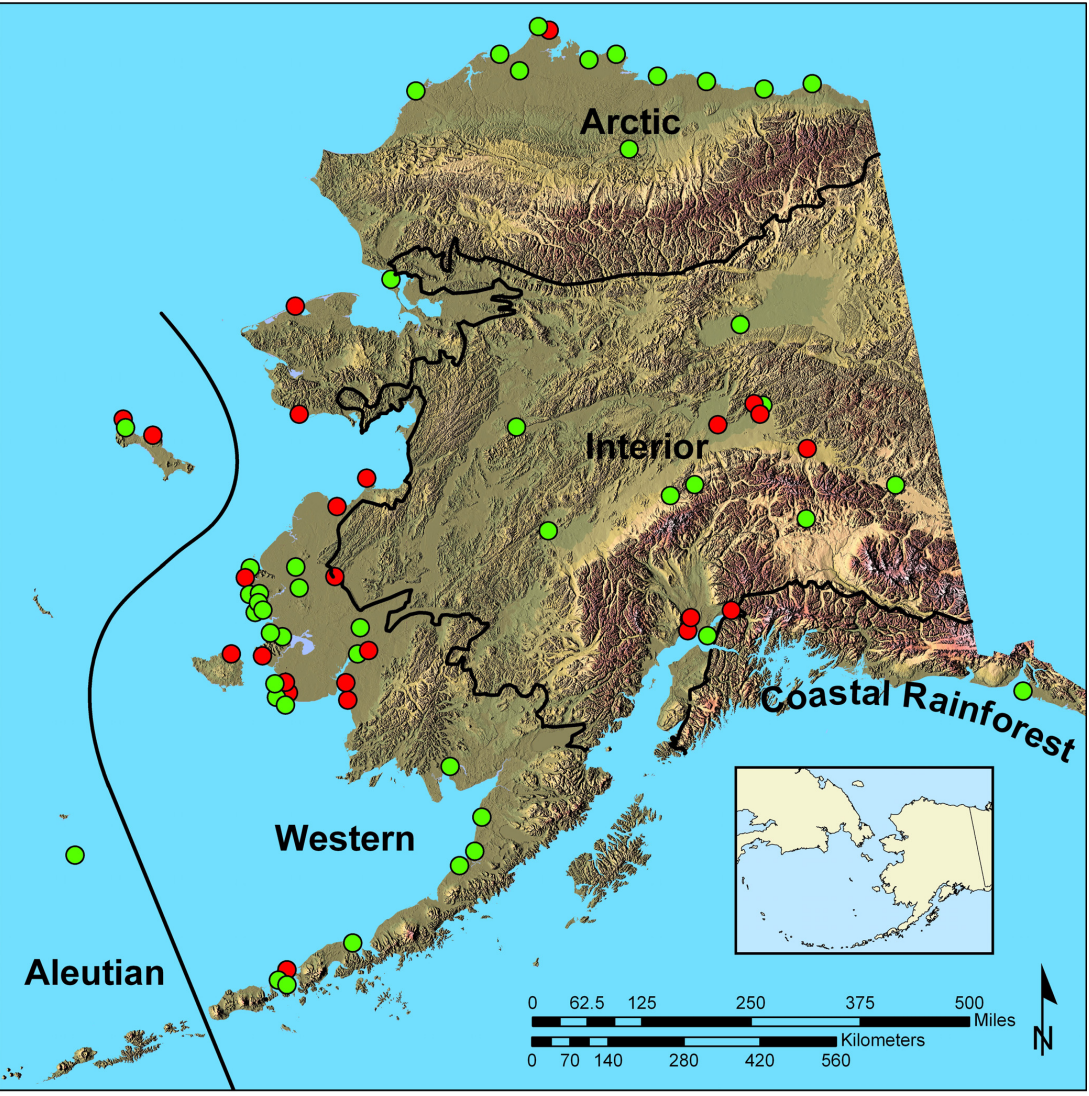
**Geographical location of sampling sites in Alaska in 2006 and 2007. **Hunter-harvest sampling locations are noted in red. Live bird surveillance sampling locations are marked in green. Habitat classifications based on Bird Conservation Regions [36,37].

We found considerable variation in overall rates of AI prevalence among birds in Alaska's five bird conservation regions (Fig. 1). The Aleutian/Bering Sea Islands region had a prevalence rate of 1.6% (n = 1,125, CI 0.9–2.5%) compared to 1.2% (n = 10,120, CI 1.1–1.5%) in Western Alaska, < 0.1% (n = 2,575, CI 0.0–0.2%) in the Arctic, 5.5% (n = 2,660, CI 4.6–6.4%) in the Interior of Alaska, and 0.6% (n = 317, CI < 0.1–2.2%) in Coastal Rainforest.

### Age and sex patterns

We restricted analyses to 10,241 birds from 97 species where both age and sex were determined. Five models assessed variation in these data by age and sex [see additional file 2: Selection results for logistic regression models used to describe variation in rRT-PCR prevalence among age and sex classifications]. The model most supported by the data included age, sex, and an interaction between age and sex. Males showed higher overall AI prevalence than females and juvenile birds had higher overall prevalence than adults. Estimated prevalence was as follows: adult females 0.6% (n = 4,300, CI 0.4–0.9%), adult males 1.3% (n = 4,015, CI 1.0–1.7%), juvenile females 5.7% (n = 967, CI 4.4–7.3%) and juvenile males 6.0% (n = 959, CI 4.6–7.6%). With the exception of one shorebird sample, all of the positive samples from juvenile birds came from waterfowl.

### Species differences

AI prevalence varied between families of birds, and among species within families. Matrix rRT-PCR positive specimens were found in 22 species of birds, with the highest rates of AI prevalence in surface-feeding ducks (Anatidae, tribe Anatini, 7.0%), followed by seabirds (Alcidae, 1.4%), gulls and terns (Laridae, 1.3%), swans (Anatidae, tribe Cygnini, 1.2%), sea ducks (Anatidae, tribe Mergini, 0.7%), and shorebirds (Scolopacidae, 0.2%) [see Additional file 1]. None of 1,927 passerines tested using rRT-PCR was positive for AI viruses.

### Seasonal differences

**Figure 2:**
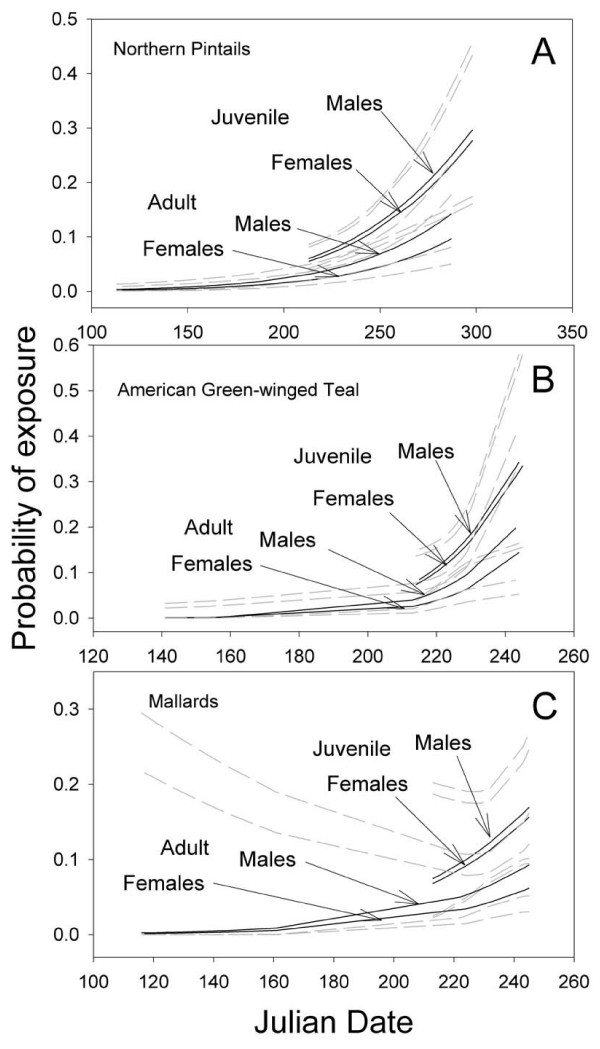
**Predicted probability of prevalence based on rRT-PCR results by logistic regression.** Lines cover the range of dates sampled for each age and sex class. Grey (dashed) lines represent the 95% confidence intervals from the logistic regression. Lines are not extrapolated beyond the range of data and therefore represent dates of sampling. A. Northern Pintails. B. American Green-winged Teal. C. Mallards.

Rates of virus prevalence were high in the spring (2.5% prior to 1 June, n = 3,959, CI 1.9–2.9%), declined in summer (0.03% 1 June – 1 Aug, n = 7,001, CI 0.01–0.04%) and increased in the autumn (3.1% after 1 Aug, n= 5,834, CI 2.6–3.5%). Three species (green-winged teal [*Anas crecca*], mallard [*A. platyrhynchos*] and northern pintail [*A. acuta*]) had sufficient samples of age, sex, and collection dates to allow assessment of trends in exposure from spring through autumn (n = 2,086). When we added covariates (date of sample collection, species, and interactions between date and species) to the best model (age, sex, and age*sex), there was strong support for inclusion of date of sampling as well as species, but little evidence of an interaction between date and species. Thus, while species have different overall rates of virus prevalence, all show similar patterns or trends across sampling dates [see additional file 3: Selection results for logistic regression models used to describe temporal and species variation in rRT-PCR prevalence among age and sex classifications]. For all 3 species, prevalence was low in the spring and increased as sampling dates progressed (Fig. 2a–c).

## Discussion

This study represents the largest single-year AI surveillance effort of wild birds in North America reported to date. Our program was implemented to monitor for possible introduction of HPAI H5N1 by migratory birds from Asia and 16,797 wild birds were sampled in Alaska. Although HPAI H5N1 was not found in any of the birds tested in 2006 and spring 2007, we detected low pathogenic AI viruses in 293 birds. Similarly, 189 low pathogenic viruses were detected by virus isolation. Because no outbreaks of HPAI H5N1 were detected during or subsequent to field sample collections, we infer that our surveillance program correctly concluded that this specific virus was not present in Alaska during our sampling. While our study was not specifically designed to compare low pathogenic avian influenza exposure rates, an examination of our findings does provide insight useful for design of future research.

### Geographic variation

AI prevalence varied among areas with the highest rate found in the interior of Alaska, and lower rates discovered along the West coast and Aleutian/Bering Sea Islands (Fig. 1). The lowest prevalence rates were found along the Arctic Coastal Plain and in Southeast Alaska. Similarly, a study of influenza A viruses in waterfowl in Alaska during the early 1990s found a higher prevalence in the interior versus coastal areas [18]. Obviously, these bird conservation regions represent broadly differing habitat types that are occupied by different species of birds. Further, these regions may represent linkages with different wintering areas within species. For example, tundra swans (*Cygnus columbianus*) sampled in the Arctic spend the winter along the East coast of North America in the Atlantic Flyway; conversely, tundra swans sampled in Western Alaska spend the winter in the western US along the Pacific and Central Flyways. Our study could not determine if the geographic variation resulted from inherent habitat characteristics that influence virus persistence and transfer, or from variation in characteristics of species or populations.

### Age and sex

Our results and those from other studies [1,19] show that AI was more prevalent in juvenile waterfowl. This pattern implies that either the adult population transmitted the viruses to young birds or that the viruses were maintained in the environment and young birds were infected following hatch [18]. The higher rate in young birds may be because they are immunologically naïve whereas adults are more resistant, particularly to viruses to which they may have previously been exposed [1].

Results of studies that examined differences in AI prevalence between sexes in birds have been inconsistent. In one case, female mallards had a greater prevalence of AI infections than males [20], more AI positives were found in males in an examination of several species [21], while a third study showed that males were more likely to test positive by H5 rRT-PCR, but not by matrix rRT-PCR, than females [19]. Other studies detected no sex difference in AI prevalence rates [22,23], however, we found differences in prevalence between sexes within age classes. The absolute difference in prevalence between sexes was greater in adults than in juveniles. For many species of waterfowl, males do not incubate eggs or rear offspring. Males of species that exhibit a sexual bias in AI prevalence rate might utilize a wider range of habitats and different foraging or roosting areas where they may encounter different groups of viruses than females. This hypothesis is supported by the greater difference between the sexes for adults compared to juveniles. At the time of our sampling, juvenile males and females would not be expected to have variation in exposure probabilities based on differing life histories.

### Species

We found considerable differences in AI prevalence among taxa but, overall, our rates are somewhat lower than found in surveillance studies conducted in Europe [22,24]. In Alaska, dabbling ducks had the highest prevalence, which is generally consistent with results from previous sampling in North America and elsewhere [3,19,25]. Detection of AI in 4 eider species, whose populations from Asia and North America co-mingle annually in Alaska, suggests they may be important hosts for the intercontinental transport of these viruses. The prevalence in Charadriiformes (0.45%) in our study is similar to the 0.42% detected in northern Europe [21], but is lower than that observed in the Chesapeake Bay region of North America (14.2%, [26]). Although several seabird species (glaucous gull [*Larus hyperboreus*], Aleutian tern [*Onychoprion aleuticus*], common murre [*Uria aalge*], and thick-billed murre [*U. lomvia*]) tested rRT-PCR positive for AI viruses, samples from only 2 species of shorebirds (bar-tailed godwit [*Limosa lapponica*] and dunlin [*Calidris alpina*]) were positive, and AI viruses were not detected in passerines.

### Season

The prevalence of AI in waterfowl is typically low on wintering areas and declines further in spring, with prevalence rates in ducks of 0.4% in late winter and 0.25% as they return to nesting areas in Canada [1,27]. In contrast, we found an unexpectedly high prevalence rate of AI (2.5% overall) in some species of spring migrants returning to Alaska. A similarly high (4.0%) prevalence was found in European ducks in the spring [22]. These data suggest that bird-to-bird transmission in waterfowl might be sufficient to perpetuate influenza viruses from year to year. In addition, high-latitude wetlands may maintain viable viruses across years [18] and infect susceptible birds upon their return. For the 3 species where we had sufficient data (i.e., pintails, mallards, green-winged teal), there were consistent non-linear trends in AI prevalence with date of sampling. As noted previously, juveniles may be more susceptible than adults and this susceptibility may explain their seasonal increase in exposure rates. However, it is not clear why the exposure rate in adults increases simultaneously. Further studies of the causes and consequences of seasonal variation in AI prevalence are needed.

### Differences between laboratory techniques

Contrary to our *a priori *expectations based on sampling, but similar to previous studies, we found higher prevalence from rRT-PCR compared to virus isolation. For example, in one study, molecular screening yielded 14.8% AI positives compared with 8.4% by virus isolation, and in another report, viruses were isolated from 60% of the molecular positives [20,22]. This disparity has been attributed to factors such as amplification from nonviable viral particles, degradation of viruses prior to egg inoculation, and the inability of some AI viruses to grow to high enough titers to be detected in eggs [20,28]. Further research is needed to identify the source of the disparity in results based on technical differences in methods.

Munster et al. [24] proposed that molecular testing is superior to the traditional technique of virus isolation for the detection of avian influenza in wild birds. We note that for large-scale surveillance programs where early detection of a possible pathogen is of paramount importance, molecular detection techniques can be readily scaled to accommodate large sample numbers. However, as noted by Munster et al. [24], even under ideal sample collection, specimen transport, and storage conditions, only 33.5% of their rRT-PCR test-positive samples resulted in virus isolation. In this study, viruses were isolated from 45% of the rRT-PCR positive samples. While some of the differences between rRT-PCR and traditional virus isolation may be due to low viral titer, inactivated or non-infectious viruses, many (32%, n = 61) of our virus isolates were from matrix rRT-PCR test-negative samples. Various species of wild birds have different diets that may lead to varying levels of non-specific inhibitors present in the cloacal swab samples. Future wild bird surveillance programs should consider the use of rRT-PCR methods with internal controls to monitor for the effects of non-specific inhibition [29]. Until the issue of rRT-PCR-negative but virus-isolation-positive samples can be resolved, we suggest that surveillance programs in wild birds should comprise a combination of molecular and traditional virus isolation methods to provide a more comprehensive assessment of the avian influenza viruses in wild birds.

### Comparison with other studies

The sources of variation noted above complicate exposure rate comparisons among studies. For example, our study found that AI prevalence, as indicated by positive matrix gene rRT-PCR test, was detected in 1.7% of the birds tested which contrasts with the 0.06% prevalence reported by Winker et al. [12], who tested 8,254 birds of 64 species in Alaska between 1998–2004. Conversely, Runstadler et al. [20] found an AI prevalence of 25.6% but only sampled 4 species of dabbling ducks, in August, at one location in interior Alaska. A recent study in Canada found AI positives in 37% of 17 species of ducks (90% of which were dabbling ducks) sampled primarily in August and September in several provinces [19]. As our results indicate, differences in prevalence between studies may be due to factors such as species composition, sex and age distribution, timing and location of sampling, as well as differences in sampling and detection methods. Thus, direct comparison and interpretation of simple prevalence rates are likely uninformative.

## Conclusion

Our large-scale surveillance study has provided a comprehensive analysis of the status of HPAI H5N1 in Alaska, where the virus had a high likelihood of first appearing in North America if introduced by migratory birds. The detailed ornithological and virological data gathered broadens our understanding of the ecology of AI in wild birds. Our data indicate that AI prevalence varied among locations, species, ages, sexes and through time. Given the patterns detected, we caution against simple comparisons among virus prevalence rates that do not control for these sources of variation. Finally, our data indicate that rRT-PCR results and virus isolation tend to differ regarding the prevalence of avian influenza viruses detected; thus, further study will be required to determine the overall best protocol for surveillance sampling as well as more detailed study of AI viruses in general. Continued surveillance under this strategy over multiple years will allow for a better understanding of the role wild birds play in the intercontinental transmission of AI, including HPAI H5N1. We encourage others to pursue risk-based sampling for H5N1 surveillance, based on current known distributions of the virus in combination with movements of migratory birds.

## Methods

### Priority species selection, sample size, and temporal and age/sex considerations

All migratory bird species linked to both Alaska and Asia were identified. Each species was ranked based on 5 factors related to the likelihood of contracting the virus in Asia and bringing it to Alaska [17]:

1) Proportion of the Alaskan population occurring in Asia. This factor is based on numerical probability such that the more individuals within a species that winter in, or migrate through, Asia, the higher the likelihood that one or more will encounter the virus.

2) Potential for contact with a known H5N1-affected area. This factor considers if these species are using areas where HPAI H5N1 is currently known to occur. The scores for this factor are anticipated to change in future years as HPAI H5N1 continues to spread and new areas of exposure continue to be identified.

3) Habitats used in Asia in the context of exposure potential. This factor considers the habitats used by each species in the context of the probability of being exposed to avian influenza. For example, long-tailed ducks (*Clangula hyemalis)* winter in areas where HPAI H5N1 is known to occur, but these birds primarily utilize near shore marine habitats reducing their probability of exposure. Conversely, northern pintails winter in areas known to have HPAI H5N1 and utilize freshwater and agricultural habitats significantly increasing their likelihood of coming into contact with domestic fowl or domestic fowl wastes.

4) Population size in Alaska. This factor considers the total population of a species that occurs in Alaska in the summer and, similar to the first factor, is based on numerical probability and de-emphasizes rare and accidental species.

5) Probability of obtaining a representative sample of sufficient size. This factor considers the likelihood of obtaining a representative sample. Alaska is very large with numerous logistical and economic constraints on access. Locations used by large numbers of some species are ephemeral and impossible to predict. Thus, this factor represents the likelihood of successful sampling.

From these factors, we developed sampling plans for the highest-ranked species. Because virus exposure in wild birds would likely not be uniformly distributed among all populations within species, geographically isolated populations for each species were identified for sampling. Chen et al. [5] isolated H5N1 from 1.8% of waterfowl sampled in live poultry markets in China. Considering these findings, we chose a target of 200 samples from each population based on our goal to detect a virus with a minimum prevalence of 1.5% at a desired 95% statistical power [30]). AI prevalence in wild birds varies by season, an individual's physical condition, and age [1]. Thus, when possible, we sampled birds at multiple times of the year, including immediately post-migration and during metabolically stressful periods, such as reproduction and molt. Further, we sampled hatch-year birds because AI prevalence has been reported to be greater in juvenile ducks than adults [1,19,22]. 'Non-target' species were sampled opportunistically when captured in conjunction with surveillance projects. These birds overlapped in distribution with priority species and were logical candidates to test for secondary exposure.

### Field sampling of live wild birds

Birds were live-captured using a variety of techniques, sexed and aged using plumage, biometric or cloacal characteristics (See Fig. 1 for sampling locations). Sampling of migratory birds followed protocols approved by the USFWS and the USGS. Cloacal swab samples were obtained by passing a sterile Dacron^® ^swab around the interior mucosa of the cloaca. Fecal samples were collected using a sterile Dacron^® ^swab or by transfer of fecal material, but only when species identity was known for individuals flushed from nest or roost sites, observed while feeding, or captured in mist nets and briefly held in a clean container prior to banding. Cloacal swabs and/or swabs of fresh fecal material were immediately placed into a vial containing 1.5 ml of viral transport media [31], kept cool in the field, and transferred to liquid nitrogen vapor shippers (-150°C) within 24 h. Vapor shippers were transported from field locations to Anchorage, Alaska, and samples transshipped on dry ice to the USGS National Wildlife Health Center, Madison, Wisconsin, for laboratory analysis.

### Sampling of birds harvested during spring subsistence and autumn hunts

Hunter-harvested birds were sampled in the spring and autumn. Within the United States, spring subsistence hunting of migratory birds is unique to rural Alaska and provided the earliest opportunity to collect samples from birds returning from Asia. Geographic areas were selected for sampling based on recent harvest records and anecdotal information on the number of priority species harvested (Fig. 1). Stations were established in villages where hunters could present birds for sampling from 1 May until 1 July.

Autumn hunter-harvested birds were sampled to augment sample numbers in locations and for species not accessed via live bird surveillance. This also enabled us to collect samples from hatch-year birds, which make up a large proportion of the autumn harvest. Locations for autumn harvest sampling were selected based on indications of hunter use, species harvested, and logistics associated with sampling birds (Fig. 1). Sampling was conducted at check stations or by directly contacting hunters in the field between 1 August and 1 December. Cloacal swabs were collected from both spring and autumn hunter-harvested birds and handled as described above.

### Laboratory analyses

Samples were analyzed individually or pooled in the laboratory in groups of 2 to 5 by species and sampling location. Molecular detection of AI viruses was performed according to the standardized USDA National Animal Health Laboratory Network AI real time RT-PCR (rRT-PCR) protocol [17,28,32]. The individual samples in all positive pools were re-tested by matrix rRT-PCR to identify the specific sample or samples that were AI positive. Positive individual samples were further analyzed using the H5 and H7 rRT-PCR tests and all H5 and H7 positive samples were sent to the USDA National Veterinary Services Laboratory in Ames, Iowa, for confirmation and pathogenicity characterization, according to the interagency surveillance plan [17,32].

All matrix rRT-PCR test positive specimens, as well 73.6% of negative specimens, were further tested by virus isolation in embryonating eggs [33]. Negative samples for virus isolation were selected at random based on laboratory capacity. Allantoic fluids from each egg were tested for the presence of hemagglutinating viruses using chicken and turkey red blood cells. Hemagglutination-negative samples were passaged at least once and re-tested before the original samples were considered negative. Hemagglutination-positive samples were further characterized as above.

### Statistical analyses

Confidence intervals for proportions were estimated following Newcombe [34]. Logistic regression was used to examine variation in prevalence of rRT-PCR-positives among *a priori *groups and across covariates. A series of *a priori *models was developed and Akaike's Information Criterion (AIC)-based selection methods were used to identify the models most supported by the data [35]. Hierarchical analyses were performed to accommodate varying sampling intensities among species.

When comparing the results of the rRT-PCR and the virus isolation tests, we expected virus isolation to yield higher AI prevalence than rRT-PCR. This assumption was based on the fact that we tested all rRT-PCR positive samples, but only a proportion (73.6%) of the rRT-PCR negatives, by virus isolation. Accordingly, we employed 1-tailed tests under a null hypothesis that rRT-PCR exposure rates would be less than virus isolation exposure rates.

## Competing interests

None of the authors has any financial interest or conflict of interest with this article. Any use of trade, product, or firm names is for descriptive purposes only and does not imply endorsement by the US government.

## Authors' contributions

HSI led the laboratory analysis. HSI, PLF, JCF and DAR wrote the manuscript. PLF, RJD, DVD, REG, CRE, JMP, RBL, SMM, DBI, JBF, RMO, MRP, TFF, TCR developed the sampling design and directed the collection of samples. DVD and DAR coordinated field collection programs and JCP provided confirmatory virus analysis. All authors approved the final version of the manuscript.

## Supplementary Material

Additional file 1 Results of avian influenza surveillance in Alaska between May 2006 and March 2007. The total number of samples collected, the number tested by matrix rRT-PCR and virus isolation in embryonating eggs, and the number of samples positive for avian influenza by both methods is presented for each of 126 species. The rRT-PCR results were used in the statistical analyses and summaries in the text. Click here for file

Additional file 2 Selection results for logistic regression models used to describe variation in rRT-PCR prevalence among age and sex classifications. Structure and associated Akaike's Information Criterion (AIC) values for models used to describe variation in rRT-PCR virus prevalence among adults and juveniles, males and females. Click here for file

Additional file 3 Selection results for logistic regression models used to describe temporal and species variation in rRT-PCR prevalence among age and sex classifications. Structure and associated Akaike's Information Criterion (AIC) values for models used to describe variation in rRT-PCR virus prevalence across dates of sampling among adults and juveniles, males and females. Click here for file
