# Supplementary material for: Prevalence of Influenza A viruses in wild migratory birds in Alaska: Patterns of variation in detection at a crossroads of intercontinental flyways
Source: Virol J. 2008 Jun 4;5:71. doi: 10.1186/1743-422X-5-71 (PMC2435106; doi:10.1186/1743-422X-5-71)
Supplement: Additional file 1 — Results of avian influenza surveillance in Alaska between May 2006 and March 2007. The total number of samples collected, the number tested by matrix rRT-PCR and virus isolation in embryonating eggs, and the number of samples positive for avian influenza by both methods is presented for each of 126 species. The rRT-PCR results were used in the statistical analyses and summaries in the text. [file 1743-422X-5-71-S1.pdf]

Results of avian influenza surveillance in Alaska between May 2006 and March 2007.[1]

The total number of samples collected, the number tested by matrix rRT-PCR and virus isolation in embryonated eggs, and the number of samples positive for avian influenza by both methods is presented for each of 126 species. The rRT-PCR results were used in the statistical analyses and summaries in the text.

Family/Tribe	Common Name	Scientific Name	Number Sampled	Number RT-PCR Positive	Number Sampled for Virus Isolation	Number Virus Isolation Positive	RT-PCR Positive (%)	Virus Isolation Positive (%)
Anatidae/Anserini	Geese		5,194	87	4,364	32	1.67	0.73
	Greater white-fronted goose	*Anser albifrons*	1,081	40	847	12	3.70	1.42
	**Emperor goose**[2]	*Chen canagica*	685	11	669	8	1.61	1.20
	**Lesser snow goose**	*C. caerulescens*	720	17	646	4	2.36	0.62
	**Black brant**	*Branta bernicla*	2,075	8	1,596	3	0.38	0.19
	Cackling goose	*B. hutchinsii*	373	2	357	1	0.53	0.28
	Canada goose	*B. canadensis*	260	9	249	4	3.46	1.61
Anatidae/Cygnini	Swans		593	7	537	7	1.18	1.30
	Trumpeter swan	*Cygnus buccinator*	10	0	4	0	0.00	0.00
	**Tundra swan**	*C. columbianus*	583	7	533	7	1.20	1.31

Anatidae/Anatini	Surface-feeding Ducks		2,342	164	1,309	107	6.99	8.17
	Gadwall	*Anas strepera*	16	0	0	0	0.00	-
	Eurasian wigeon	*A. penelope*	1	0	1	0	0.00	0.00
	American wigeon	*A. americana*	64	0	26	0	0.00	0
	Mallard	*A. platyrhynchos*	273	25	157	25	9.16	15.92
	Blue-winged teal	*A. discors*	1	0	0	0	0.00	-
	Northern shoveler	*A. clypeata*	22	1	19	1	4.55	5.26
	**Northern pintail**	*A. acuta*	1,426	87	793	57	6.10	7.19
	Green-winged teal	*A. crecca*	539	51	313	24	9.43	7.67
Anatidae/Aythyini	Bay Ducks		29	0	24	0	0.00	0.00
	Canvasback	*Aythya valisineria*	8	0	4	0	0.00	0.00
	Redhead	*A. americana*	1	0	0	0	0.00	-
	Greater scaup	*A. marila*	1	0	1	0	0.00	0.00
	Lesser scaup	*A. affinis*	19	0	19	0	0.00	0.00
Anatidae/Mergini	Sea Ducks		2,332	16	1,716	18	0.69	1.05
	**Steller's eider**	*Polysticta stelleri*	737	6	300	3	0.81	1.00
	**Spectacled eider**	*Somateria fischeri*	348	2	293	1	0.57	0.34
	**King eider**	*S. spectabilis*	680	6	640	9	0.88	1.41
	**Common eider**	*S. mollissima*	395	1	383	4	0.25	1.04

	Harlequin duck	*Histrionicus histrionicus*	65	0	0	0	0.00	-
	Surf scoter	*Melanitta perspicillata*	2	0	2	0	0.00	0.00
	White-winged scoter	*M. fusca*	41	0	41	0	0.00	0.00
	Black scoter	*M. nigra*	10	0	10	0	0.00	0.00
	**Long-tailed duck**	*Clangula hyemalis*	52	1	46	1	1.92	2.17
	Bufflehead	*Bucephala albeola*	1	0	1	0	0.00	0.00
	Common merganser	*Mergus merganser*	1	0	1	0	0.00	0.00
Gaviidae	Loons		9	0	5	0	0.00	0.00
	Red-throated loon	*Gavia stellata*	3	0	0	0	0.00	-
	Arctic loon	*G. arctica*	1	0	1	0	0.00	0.00
	Pacific loon	*G. pacifica*	2	0	2	0	0.00	0.00
	Common loon	*G. immer*	1	0	1	0	0.00	0.00
	Yellow-billed loon	*G. adamsii*	2	0	1	0	0.00	0.00
Podicipedidae	Grebes		1	0	1	0	0.00	0.00
	Red-necked grebe	*Podiceps grisegena*	1	0	1	0	0.00	0.00
Procellariidae	Shearwaters, Petrels		1	0	1	0	0.00	0.00
	Northern fulmar	*Fulmarus glacialis*	1	0	1	0	0.00	0.00
Phalacrocoracidae	Cormorants		5	0	5	0	0.00	0.00
	Pelagic cormorant	*Phalacrocorax pelagicus*	3	0	3	0	0.00	0.00

	Red-faced cormorant	*P. urile*	2	0	2	0	0.00	0.00
Gruidae	Cranes		161	0	131	0	0.00	0.00
	**Sandhill crane**	*Grus canadensis*	161	0	131	0	0.00	0.00
Charadriidae	Lapwings, Plovers		140	0	122	0	0.00	0.00
	Black-bellied plover	*Pluvialis squatarola*	73	0	63	0	0.00	0.00
	American golden plover	*P. dominica*	15	0	14	0	0.00	0.00
	**Pacific golden plover**	*P. fulva*	42	0	35	0	0.00	0.00
	Golden plover spp.	*P. spp.*	6	0	6	0	0.00	0.00
	Semipalmated plover	*Charadrius semipalmatus*	4	0	4	0	0.00	0.00
Scolopacidae	Sandpipers, Phalaropes		3,040	5	2,402	1	0.16	0.04
	Greater yellowlegs	*T. melanoleuca*	1	0	1	0	0.00	0.00
	Whimbrel	*Neumenius phaeopus*	1	0	1	0	0.00	0.00
	Bristle-thighed curlew	*N. tahitiensis*	9	0	9	0	0.00	0.00
	**Bar-tailed godwit**	*Limosa lapponica*	210	3	167	0	1.43	0.00
	**Ruddy turnstone**	*Arenaria interpres*	30	0	27	0	0.00	0.00
	Black turnstone	*A. melanocephala*	2	0	2	0	0.00	0.00
	**Red knot**	*Calidris canutus*	79	0	71	0	0.00	0.00
	Semipalmated sandpiper	*C. pusilla*	212	0	108	0	0.00	0.00
	Western sandpiper	*C. mauri*	128	0	52	0	0.00	0.00

	Baird’s sandpiper	*C. bairdii*	1	0	1	0	0.00	0.00
	**Pectoral sandpiper**	*C. melanotos*	580	0	549	1	0.00	0.18
	**Sharp-tailed sandpiper**	*C. acuminate*	225	0	192	0	0.00	0.00
	**Rock sandpiper**	*C. ptilocnemis*	173	0	156	0	0.00	0.00
	**Dunlin**	*C. alpina*	897	2	682	0	0.22	0.00
	Stilt sandpiper	*C. himantopus*	3	0	3	0	0.00	0.00
	**Buff-breasted sandpiper**	*Tryngites subruficollis*	92	0	81	0	0.00	0.00
	Ruff	*Philomachus pugnax*	6	0	6	0	0.00	0.00
	**Long-billed dowitcher**	*Limnodromus scolopaceus*	165	0	157	0	0.00	0.00
	Wilson’s snipe	*Gallinago delicata*	9	0	5	0	0.00	0.00
	Red-necked phalarope	*Phalaropus lobatus*	38	0	18	0	0.00	0.00
	Red phalarope	*P. fulicarius*	179	0	114	0	0.00	0.00
Laridae	Gulls, Terns		453	6	358	7	1.32	1.96
	Mew gull	*Larus canus*	5	0	5	0	0.00	0.00
	Herring gull	*L. argentatus*	4	0	3	0	0.00	0.00
	**Glaucous gull**	*L. hyperboreus*	138	5	45	4	3.60	8.89
	Sabine’s gull	*Xema sabini*	1	0	0	0	0.00	-
	Black-legged kittiwake	*Risa tridactyla*	2	0	2	0	0.00	0.00
	**Aleutian tern**	*Onychoprion aleuticus*	302	1	302	3	0.33	0.99

	Arctic tern	*Sterna paradisaea*	1	0	1	0	0.00	0.00
Stercorariidae	Jaegers		6	0	5	0	0.00	0.00
	Pomarine jaeger	*Stercorarius pomarinus*	4	0	3	0	0.00	0.00
	Long-tailed jaeger	*S. longicaudus*	2	0	2	0	0.00	0.00
Alcidae	Auks, Murres, Puffins		562	8	562	16	1.42	2.85
	Common murre	*Uria aalge*	76	1	76	2	1.32	2.63
	Thick-billed murre	*U. lomvia*	235	7	235	10	2.98	4.26
	Parakeet auklet	*Aethia psittacula*	8	0	8	0	0.00	0.00
	Least auklet	*A. pusilla*	30	0	30	0	0.00	0.00
	Crested auklet	*A. cristatella*	207	0	207	4	0.00	1.93
	Horned puffin	*Fratercula corniculata*	2	0	2	0	0.00	0.00
	Tufted puffin	*F. cirrhata*	4	0	4	0	0.00	0.00
Strigidae	Owls		1	0	0	0	0.00	-
	Short-eared owl	*Asio flammeus*	1	0	0	0	0.00	-
Picidae	Woodpeckers		1	0	0	0	0.00	-
	Northern flicker	*Colaptes auratus*	1	0	0	0	0.00	-
Tyrannidae	Tyrant Flycatchers		5	0	0	0	0.00	-
	Alder flycatcher	*Empidonax alnorum*	5	0	0	0	0.00	-
Laniidae	Shrikes		2	0	1	0	0.00	0.00

	Northern shrike	*Lanius excubitor*	2	0	1	0	0.00	0.00
Corvidae	Crows, Jays		5	0	3	0	0.00	0.00
	Gray jay	*Perisoreus canadensis*	2	0	0	0	0.00	-
	Common raven	*Corvus corax*	3	0	3	0	0.00	0.00
Alaudidae	Larks		1	0	1	0	0.00	0.00
	Horned lark	*Eremophila alpestris*	1	0	1	0	0.00	0.00
Hirundinidae	Swallows		2	0	0	0	0.00	-
	Tree swallow	*Tachycineta bicolor*	1	0	0	0	0.00	-
	Cliff swallow	*Petrochelidon pyrrhonota*	1	0	0	0	0.00	-
Paridae	Chickadees		1	0	0	0	0.00	-
	Black-capped chickadee	*Poecile atricapillus*	1	0	0	0	0.00	-
Certhiidae	Creepers		1	0	0	0	0.00	-
	Brown creeper	*Certhia americana*	1	0	0	0	0.00	-
Regulidae	Kinglets		2	0	0	0	0.00	-
	Ruby-crowned kinglet	*Regulus calendula*	2	0	0	0	0.00	-
Sylviidae	Old World Warblers		769	0	409	0	0.00	0.00
	**Arctic warbler**	*Phylloscopus borealis*	769	0	409	0	0.00	0.00
Turdidae	Thrushes		302	0	137	0	0.00	0.00
	Bluethroat	*Luscinia svecica*	12	0	6	0	0.00	0.00

	Northern wheatear	*Oenanthe oenanthe*	10	0	1	0	0.00	0.00
	**Gray-cheeked thrush**	*Catharus minimus*	229	0	124	0	0.00	0.00
	Swainson's thrush	*C. ustulatus*	20	0	0	0	0.00	-
	Hermit thrush	*C. guttatus*	10	0	1	0	0.00	0.00
	American robin	*Turdus migratorius*	11	0	5	0	0.00	0.00
	Varied thrush	*Ixoreus naevius*	10	0	0	0	0.00	-
Motacillidae	Wagtails, Pipits		306	0	217	0	0.00	0.00
	**Eastern yellow wagtail**	*Motacilla tschutschensis*	304	0	216	0	0.00	0.00
	American pipit	*Anthus rubescens*	2	0	1	0	0.00	0.00
Parulidae	Wood warblers		135	0	27	0	0.00	0.00
	Orange-crowned warbler	*Vermivora celata*	44	0	2	0	0.00	0.00
	Yellow warbler	*Dendroica petechia*	7	0	7	0	0.00	0.00
	Yellow-rumped warbler	*D. coronata*	11	0	0	0	0.00	-
	Bay-breasted warbler	*D. castanea*	1	0	0	0	0.00	-
	Blackpoll warbler	*D. striata*	3	0	1	0	0.00	0.00
	Northern waterthrush	*Seiurus noveboracensis*	27	0	13	0	0.00	0.00
	Wilson's warbler	*Wilsonia pusilla*	42	0	4	0	0.00	0.00
Emberizidae	Emberizids		329	0	90	1	0.00	1.11
	American tree sparrow	*Spizella arborea*	96	0	29	0	0.00	0.00

	Savannah sparrow	*Passerculus sandwichensis*	92	0	30	0	0.00	0.00
	Fox sparrow	*Passerella iliaca*	26	0	15	0	0.00	0.00
	Lincoln's sparrow	*Melospiza lincolnii*	20	0	0	0	0.00	-
	White-crowned sparrow	*Zonotrichia leucophrys*	49	0	5	0	0.00	0.00
	Golden-crowned sparrow	*Z. atricapilla*	20	0	3	0	0.00	0.00
	Dark-eyed junco	*Junco hyemalis*	16	0	0	0	0.00	-
	Lapland longspur	*Calcarius lapponicus*	8	0	6	0	0.00	0.00
	Snow bunting	*Plectrophenax nivalis*	2	0	2	1	0.00	50.00
Fringillidae	Finches		67	0	15	0	0.00	0.00
	Brambling	*Fringilla montifringilla*	1	0	1	0	0.00	0.00
	Pine grosbeak	*Pinicola enucleator*	3	0	0	0	0.00	-
	White-winged crossbill	*Loxia leucoptera*	1	0	0	0	0.00	-
	Common redpoll	*Carduelis flammea*	52	0	9	0	0.00	0.00
	Hoary redpoll	*C. hornemanni*	10	0	5	0	0.00	0.00
TOTALS			16,797	293	12,442	189	1.74	1.52

[1]Additional details regarding age, sex, and geographic composition of samples can be found in USFWS/USGS: Sampling for highly pathogenic Asian H5N1 avian influenza in migratory birds in Alaska: results of 2006 field season. Progress Report, U. S. Fish and Wildlife Service (Region 7, Alaska) and U. S. Geological Survey, Alaska Science Center, Anchorage Alaska. [http://Alaska.usgs.gov/science/biology/avian_influenza/pdfs/USFWS-USGS_2006_AK_HPAI_H5N1_Sampling_Accom_Rep.pdf].

[2]Common names of birds in bold font were priority species for sampling.
