# Supplementary material for: Prevalence of Influenza A viruses in wild migratory birds in Alaska: Patterns of variation in detection at a crossroads of intercontinental flyways
Source: Virol J. 2008 Jun 4;5:71. doi: 10.1186/1743-422X-5-71 (PMC2435106; doi:10.1186/1743-422X-5-71)
Supplement: Additional file 2 — Selection results for logistic regression models used to describe variation in rRT-PCR prevalence among age and sex classifications. Structure and associated Akaike's Information Criterion (AIC) values for models used to describe variation in rRT-PCR virus prevalence among adults and juveniles, males and females. [file 1743-422X-5-71-S2.pdf]

Selection results for logistic regression models used to describe variation in rRT-PCR prevalence among age and sex classifications.

Model Structure [a]	# parameters [a]	AIC [b]	ΔAIC [c]	AIC weight [d]
Sex, age sex*age	4	1795.3	0	0.675
Sex, age	3	1797.4	2.1	0.245
Age	2	1799.6	4.3	0.079
Sex	2	1945.9	150.6	<0.001
Constant	1	1948.9	153.6	<0.001

a. n=10,241.

b. Number of parameters in each model.

c. Akaike's Information Criterion value.

d. Difference in AIC value for each model relative to the lowest AIC for each species. Δ AIC = 0 indicates the most parsimonious model for each species.

e. Weight of evidence that a model represents the true best model considered.
