# Supplementary material for: Prevalence of Influenza A viruses in wild migratory birds in Alaska: Patterns of variation in detection at a crossroads of intercontinental flyways
Source: Virol J. 2008 Jun 4;5:71. doi: 10.1186/1743-422X-5-71 (PMC2435106; doi:10.1186/1743-422X-5-71)
Supplement: Additional file 3 — Selection results for logistic regression models used to describe temporal and species variation in rRT-PCR prevalence among age and sex classifications. Structure and associated Akaike's Information Criterion (AIC) values for models used to describe variation in rRT-PCR virus prevalence across dates of sampling among adults and juveniles, males and females. [file 1743-422X-5-71-S3.pdf]

Selection results for logistic regression models used to describe temporal and species variation in rRT-PCR prevalence among age and sex classifications.

Model Structure [a]	# parameters [b]	AIC [c]	ΔAIC [d]	AIC weight [e]
Sex, age, sex*age, date, species	7	1054.1	0.0	0.696
Sex, age, sex*age, date, species, species*date	9	1055.8	1.7	0.294
Sex, age, sex*age, date	5	1062.6	8.50	0.010
Sex, age, sex*age, species	6	1079.9	25.8	<0.001
Sex, age, sex*age	4	1084.2	30.1	<0.001
Constant	1	1111.1	57	<0.001

a. n=2,086.
b. Number of parameters in each model.
c. Akaike's Information Criterion value.
d. Difference in AIC value for each model relative to the lowest AIC for each species. Δ AIC = 0 indicates the most parsimonious model for each species.
e. Weight of evidence that a model represents the true best model considered.
